# Embryonic sympathoblasts transiently express TrkB *in vivo *and proliferate in response to brain-derived neurotrophic factor *in vitro*

**DOI:** 10.1186/1471-213X-7-10

**Published:** 2007-02-19

**Authors:** Jennifer A Straub, Giselle L Saulnier Sholler, Rae Nishi

**Affiliations:** 1 Department of Anatomy and Neurobiology, University of Vermont College of Medicine, HSRF 406, 149 Beaumont Ave, Burlington, VT, 05405-0075, USA; 2 Department of Pediatrics, University of Vermont College of Medicine, Given E203, 89, Beaumont Avenue, Burlington, VT, 05405-0068, USA

## Abstract

**Background:**

Nerve growth factor and neurotrophin-3 are involved in the development of sympathetic neurons; however, whether brain derived neurotrophic factor also plays a role is not known. The purpose of this study was to determine whether BDNF and its receptor, TrkB, are expressed during the development of paravertebral sympathetic ganglia *in vivo *and to determine the effect of BDNF *in vitro*.

**Results:**

As neural crest cells coalesce to form sympathetic ganglia, TrkB-positive cells are seen in both chicken and mouse embryos. In chicken embryos, TrkB-expressing cells first appear at Hamburger-Hamilton Stage (St) 27 and they co-express HNK-1, confirming that they are migrating neural crest cells. The TrkB-positive cells lack neural markers at this stage; however, they migrate with other neurally differentiating cells that are TrkA and TrkC-positive. By St. 29/30, TrkB-positive cells begin to express the neural specific markers Hu C/D and Islet-1; eventually, all TrkB positive cells commence neural differentiation. By St. 34, TrkB and TrkC staining are lost. BDNF transcript expression parallels that of TrkB. In the mouse, TrkB-positive cells surround newly formed sympathetic ganglia and a small number of TrkB positive cells that co-express tyrosine hydroxylase are seen within ganglia between E13.5-15. In cell culture, many cells from St. 29–30 chicken lumbar sympathetic ganglia express neural markers and are dividing, indicating that they are sympathoblasts. Sympathoblasts and neurons require both nerve growth factor and neurotrophin-3 for survival. BDNF increases the number of cells expressing neural markers in culture by increasing number of cells that incorporate bromodeoxyuridine. In contrast, most TrkB-positive sympathetic cells *in vivo *are not actively proliferating between E6–E8.

**Conclusion:**

Developing paravertebral sympathetic ganglia in avian and murine embryos contain a subpopulation of sympathoblasts that transiently express TrkB and ultimately commence neuronal differentiation. These TrkB expressing sympathoblasts are not actively dividing *in vivo*; yet, when placed *in vitro*, will divide in response to BDNF. This suggests that the availability of BDNF in vivo fails to reach a threshold necessary to induce proliferation. We suggest that excess TrkB stimulation of sympathoblasts *in vivo *may lead to the genesis of neuroblastoma.

## Background

Neural crest cells destined to become paravertebral sympathetic neurons proliferate and differentiate during migration and gangliogenesis. In chicken embryos, migrating neural crest cells express catecholamines at Hamburger/Hamilton Stage (St.) 19, and these cells form the primary sympathetic chain dorsolateral to the aorta at St. 22 (E3.5) [1]. Between St. 23 (E4) and St. 28 (E6), these cells disperse and undergo a secondary migration to form the paravertebral sympathetic chain that resides ventral to the spinal cord and dorsal root ganglion [1]. After ganglia coalesce, sympathoblasts express markers of neuronal differentiation, such as Q211 and tyrosine hydroxylase (TH), at a time when they also incorporate [^3^H]-thymidine [2]. Time lapse photography has shown that cultured E15.5–E16.5 sympathetic neurons from rat embryos extend axons while they divide [3-5]. Although proliferation appears to be an important process to expand the sympathetic neuron population during differentiation, the mechanisms that guide sympathoblast proliferation have not been identified.

The development of sympathetic neurons is guided by neurotrophins. Neurotrophin-3 (NT-3) binds to its receptor, TrkC, to promote the survival of cultured sympathoblasts from early lumbar paravertebral ganglia [6]. Nerve growth factor (NGF) signals through its receptor, TrkA, to promote the survival of sympathetic neurons upon target innervation [7]. There are severe sympathetic defects in the superior cervical ganglion of individual NT-3 and NGF knockout mice [8-10]. Furthermore, there is no additional cell death in the superior cervical ganglion of NT-3 and NGF double knockout mouse embryos, suggesting that all of the neurons are dependent on both neurotrophins for survival [11]. There is also an increase in sympathetic neuron cell death in TrkA knockout mice [12]. However, in TrkB and BDNF knockout mice, there is no apparent phenotype in the superior cervical ganglion, and there is little evidence that TrkB or BDNF is expressed in sympathetic ganglia. Thus, it is generally thought that TrkB and BDNF have little or no roles in guiding the development of sympathetic neurons.

In addition to their developmental functions, neurotrophin receptors regulate cell behavior in neuroblastoma, a tumor found in sympathetic ganglia and adrenal medulla. Tumors that express TrkA often spontaneously regress, while those that express TrkB and its ligand, brain-derived neurotrophic factor (BDNF), grow aggressively, are invasive, and fail to respond to chemotherapeutic agents [13]. The presence of TrkA in neuroblastoma tumors is consistent with its expression in developing sympathetic neurons, and suggests that regressive neuroblastoma tumors arise from early sympathetic neurons that express TrkA. The function of TrkB in early sympathetic development is unknown, which makes understanding the etiology of aggressive neuroblastoma tumors difficult. Based on its function in neuroblastoma tumors, we hypothesize that BDNF and TrkB expression in differentiating sympathoblasts is responsible for expanding the neuronal population through proliferation.

We sought to determine whether BDNF and TrkB are involved in sympathetic development. We report that during early embryonic development, TrkB is expressed in a subset of differentiating sympathoblasts in both avian and murine embryos. We also find that BDNF promotes the proliferation of TrkB-positive sympathoblasts in cell culture. However, the majority of TrkB positive cells in vivo fail to take up bromodeoxyuridine (BrdU) over a 24 hr period, suggesting that endogenous BDNF concentrations do not reach a threshold necessary to stimulate proliferation of sympathoblasts. Shortly after all of the TrkB positive cells commence neuronal differentiation, TrkB immunoreactivity is lost. These results suggest that prolonged expression and/or activation of TrkB signaling at these early stages may be an early event triggering the formation of neuroblastoma.

## Results

### TrkB is expressed during migration of neural crest cells to sympathetic ganglia

We first determined whether TrkB is expressed in neural crest-derived cells in the region ventral to the spinal cord and dorsal root ganglia where sympathetic ganglia coalesce between Hamburger/Hamilton Stages (St.) 25–28/29. To identify cells that have commenced neuronal differentiation, transverse sections of the lumbar spinal column region were stained with antibodies against Hu C/D [14], a neuronal-specific RNA-binding protein, or Islet-1, a transcription factor found in sympathetic neurons [15]. We found that Hu C/D and Islet-1 are expressed in the same cells both *in vivo *and *in vitro *throughout sympathetic development. In experiments done between St. 25 and 28, Islet-1 staining appeared weaker than Hu C/D staining, and thus we used Hu C/D to identify differentiating neurons at these stages. At later stages, Islet-1 was used to facilitate the identification of neurons because of the nuclear location of its immunoreactivity.

**Figure 1:**
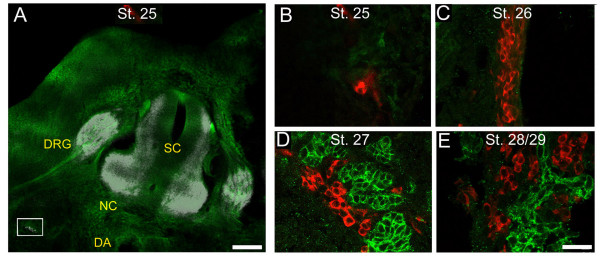
**TrkB-expressing cells surround neurons during sympathetic gangliogenesis**. Chicken embryos at St. 25, 26, 27, and 28/29 were sectioned through the trunk and the patterns of Hu C/D-(white or red) and TrkB-(green) immunoreactivity were determined. (A) Low and (B) high power images demonstrating that at St. 25 (early E5) very few Hu C/D-positive cells are found in the region where sympathetic ganglia form, ventral to the spinal cord (SC) and dorsal root ganglion (DRG) and lateral to the dorsal aorta (DA). (C) At St. 26, migrating Hu C/D-positive cells are present, but TrkB is still not expressed. (D) At St. 27, TrkB-positive cells "cluster" around Hu C/D-expressing cells, although TrkB is not expressed in the neuronal cells. (E) Sympathetic ganglia coalesce at St. 28/29, and TrkB-expressing cells remain adjacent to Hu C/D expressing cells. The box in (A) indicates the region where we obtained high power images. Calibration bars: Low power image: 200 μ; high power images: 20 μ.

**Figure 2:**
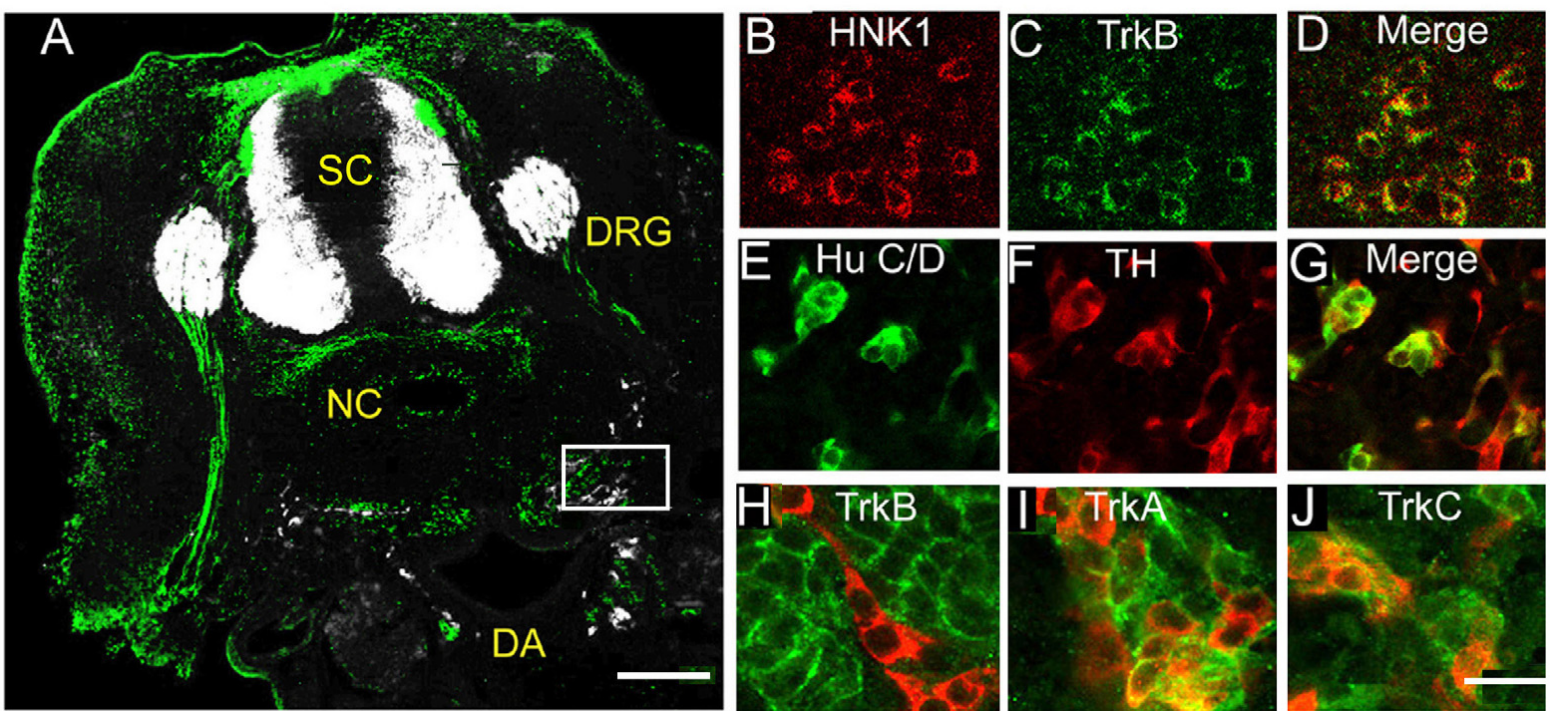
**TrkB-expressing neural crest cells migrate with sympathetic neurons at stage 27, or late E5**. (A) Transverse section of St. 27 (late E5) chick embryo spinal column region. At this stage, a stream of Hu C/D-positive cells (white) and TrkB-positive cells (green) are found in the region surrounding the dorsal aorta (DA) and ventral to the neural tube (NT), dorsal root ganglion (DRG), and notochord (NC). (B, C, D) St. 27 sections stained for TrkB (green) and a marker of neural crest cells, HNK-1 (red). In migrating clusters of TrkB-positive cells, HNK-1 is co-expressed. (E, F, G) St. 27 sections stained for Hu C/D (green) and tyrosine hydroxylase (red). All of the migrating neurons and some of the surrounding cells express tyrosine hydroxylase. (H) TrkB (green) is only expressed in cells adjacent to Hu C/D-positive cells (red), (I, J) TrkA and TrkC (both green) are expressed in both Hu C/D-positive (red) migrating cells and in surrounding cells. The box in (A) indicates the region where we obtained high power images. Calibration bars: Low power image: 200 μ; high power images: 20 μ.

**Figure 3:**
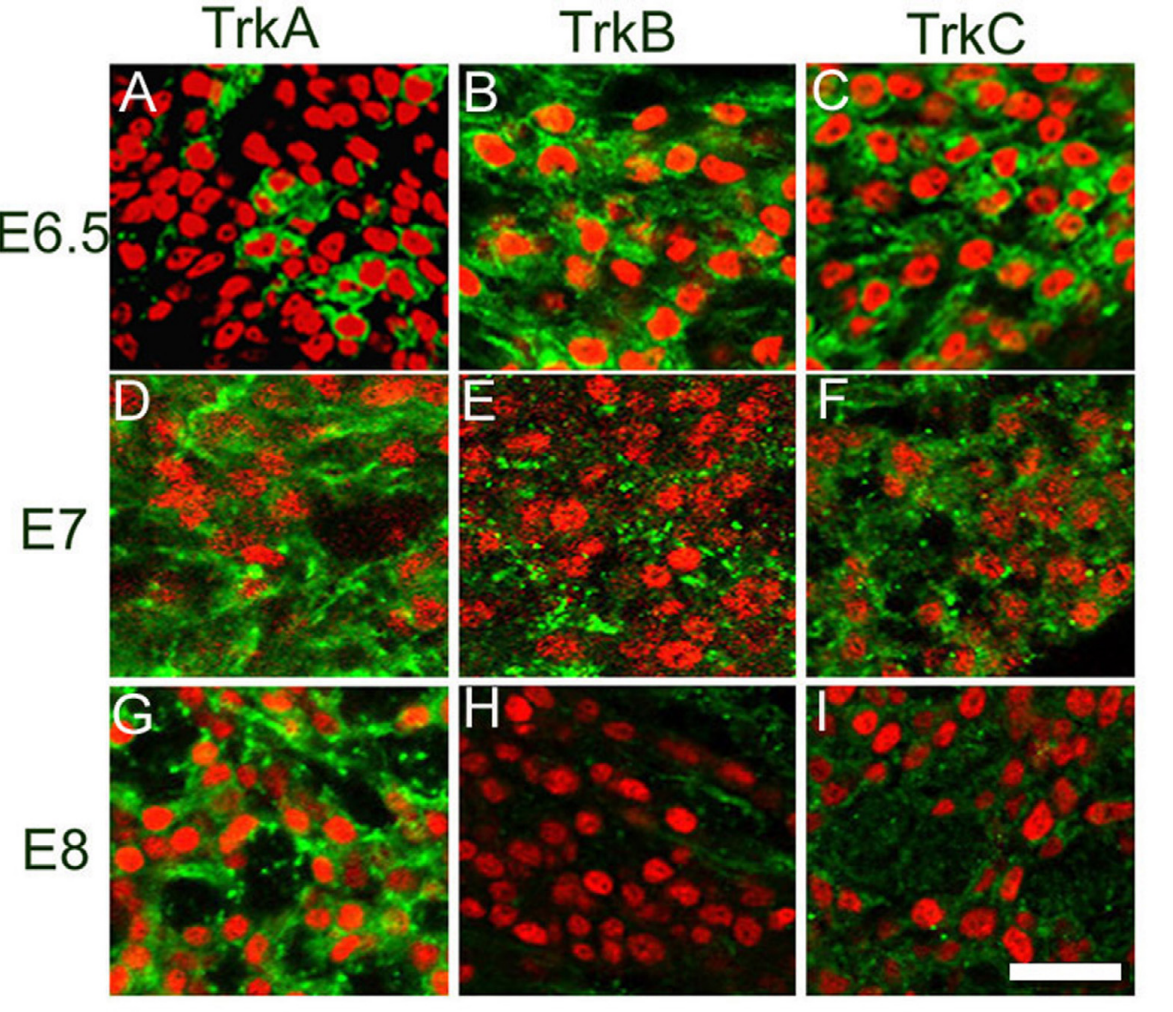
**Developmental regulation of Trk receptors in sympathetic ganglia**. Transverse sections of St 29/30 (E6.5), St. 31 (E7), and St. 34 (E8) sympathetic ganglia were labeled for Islet-1 (red) and TrkA, TrkB, or TrkC (green). The number of Islet-1-positive cells that express TrkA increases between St. 29/30 (E6.5) and St. 31 (E7), and all of the neurons express TrkA at St. 34 (E8) (A, D, G). Islet-1 positive cells express TrkB and TrkC at St. 29/30 (E6.5) (B, C). TrkB and TrkC expression appeared dispersed at St. 31 (E7) (E, F), and expression was no longer detectable at St. 34 (E8) (H, I). Calibration bar: 20 μ.

Cells expressing Hu C/D are first detected at St. 25 ventral to the spinal cord and dorsal root ganglion and lateral to the dorsal aorta (Figure 1A, 1B). By St. 26, the number of cells that express Hu C/D in this region increases dramatically (Figure 1C). TrkB-expressing cells first appear at St. 27 in the same region and are adjacent to Hu C/D-positive cells (Figure 1D, 2A, 2H). TrkB-positive cells co-localize with a neural crest marker, HNK-1 (Figures 2B, 2C, 2D). The Hu C/D-positive cells in this region are likely to be sympathetic neurons, since they appear in the region where sympathetic ganglia form and express tyrosine hydroxylase, a rate-limiting enzyme in the synthesis of catecholamines (Figures 2E, 2F, 2G). At St. 28/29, the cells begin to coalesce ventral to the dorsal root ganglion and the Hu C/D-positive cells and TrkB-positive cells remain as two separate cell populations (Figure 1E); however, shortly afterwards, all of the TrkB-positive cells begin to express Islet-1 (Figure 3B) and Hu C/D (data not shown).

### Developmental regulation of TrkA, TrkB, TrkC, and BDNF expression

**Figure 4:**
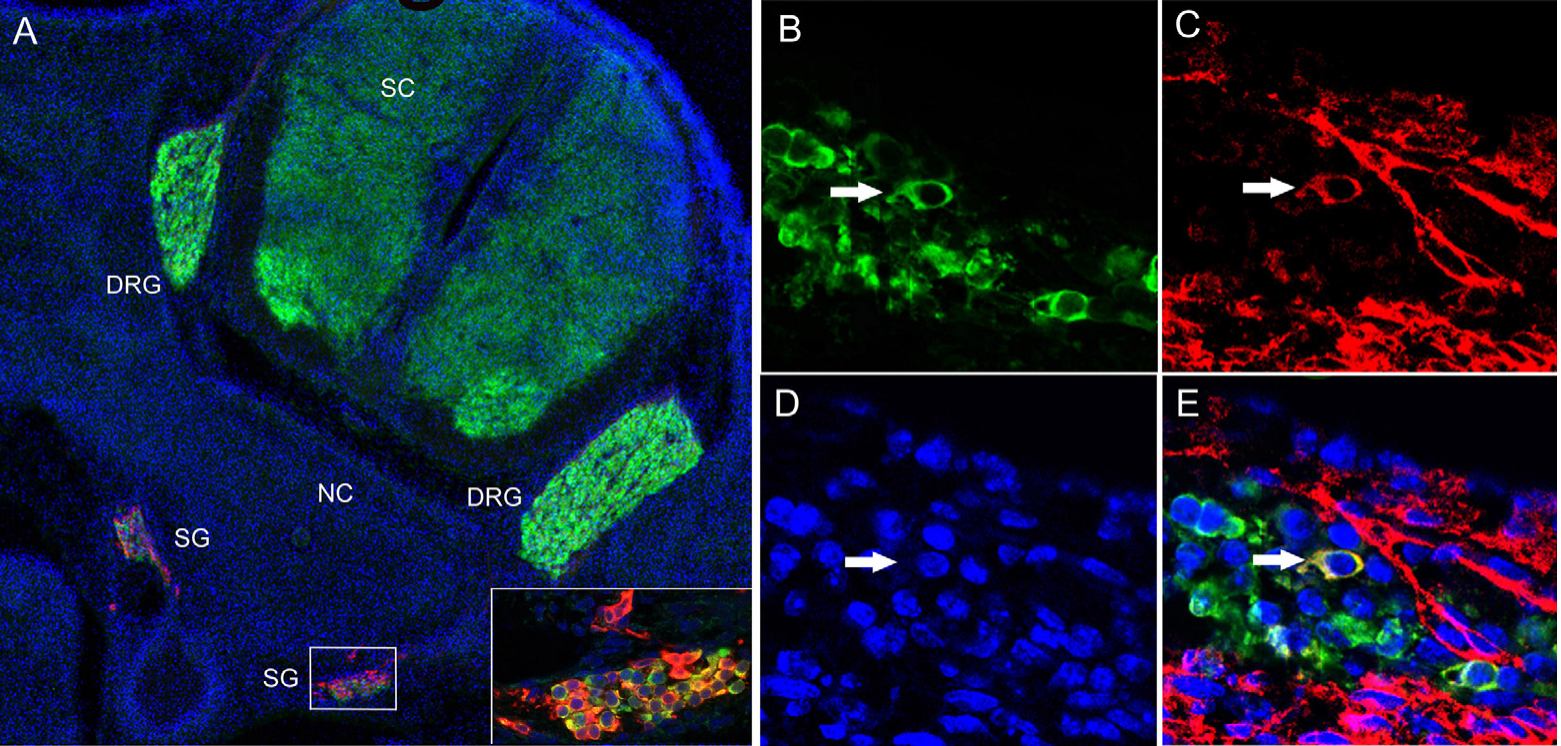
**A subpopulation of cells in developing mouse sympathetic ganglia expresses TrkB**. (A) Transverse sections of E13.5 mouse embryos were cut and stained for TH (red) and Hu C/D (green) to locate developing ganglia. The ganglion shown in the box is shown at higher magnification in the inset. Different subsets of cells express TH, Hu C/D, or both markers. Sections taken from another ganglion stained with (B) TH (green), (C) TrkB (red), (D) Hoechst dye, and the overlay of all three photos. Arrow points to a cell that co-expresses TH and TrkB.

In contrast to TrkB, the other neurotrophin receptors, TrkA and TrkC, are co-expressed in both Hu C/D-positive and Hu C/D-negative cells at St. 27 (Figures 2I, 2J). We find that approximately 30% of the Islet-1-positive cells express TrkA (Figure 3A), while 50% express TrkB (Figure 3B) and 100% express TrkC (Figure 3C) at St. 29/30 (E6.5). Thus, all developing neurons express TrkC in combination with either TrkA or TrkB. By St. 31 (E7), the number of TrkA-positive, Islet-1-positive cells increases to 100% (Figure 3D) and immunoreactivities for both TrkB and TrkC appear dispersed (Figure 3E, 3F). By St. 34 (E8), TrkA expression is well-sustained (Figure 3G) and TrkB and TrkC immunoreactivities are lost (Figure 3H, 3I). We also examined the early development of murine sympathetic ganglia (Figure 4). At E13, the newly formed lumbar sympathetic ganglia can be observed ventral to the spinal cord and notochord by their staining for Hu C/D and TH (Figure 4A). TrkB-positive cells can be seen surrounding developing ganglia, as well as in occasional cells within the ganglia (Figure 4C, D). These TrkB-positive cells within the ganglia co-express TH and are seen at a frequency of 1–2 cells per section starting at E13 (Figure 4A–D) and are still present at E15.5 (data not shown).

**Figure 5:**
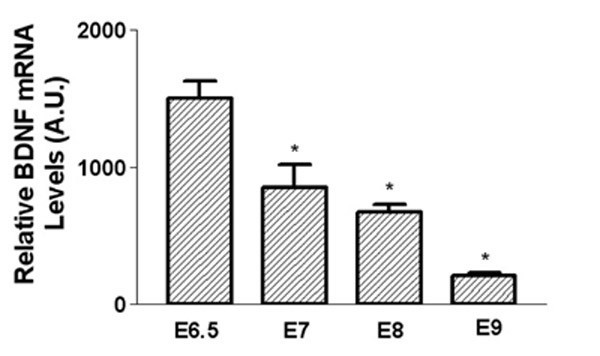
**BDNF mRNA expression is developmentally regulated in embryonic sympathetic ganglia**. Lumbar sympathetic ganglia were removed from chick embryos at St. 29/30 (E6.5), St. 31 (E7), St. 34 (E8), and E9 and rapidly frozen on dry ice. RNA was isolated and cDNA was reverse-transcribed using oligo-dT. Real-time PCR amplification for chicken BDNF was performed using TaqMan probes and normalizing target values to the constitutively expressed RNA encoding chick ribosomal binding protein S-17 (Chrps). BDNF levels are highest at St. 29/30 (E6.5) and levels steadily decline at the later stages of development, paralleling the expression pattern of TrkB. Data represent the mean ± SEM from triplicate samples. Similar results were obtained in two separate experiments. *Denotes statistical significance (p < 0.05), one-way ANOVA.

In neuroblastoma cells, BDNF is co-expressed with TrkB, suggesting that autocrine stimulation is a means by which proliferation is sustained in the transformed cells. To test whether BDNF, the ligand for TrkB, was present in embryonic chick sympathetic ganglia, we used quantitative real-time PCR with TaqMan probes to determine the relative abundance of BDNF transcripts in total RNA extracted from lumbar sympathetic ganglia at St. 29/30 (E6.5), St. 31 (E7), St. 34 (E8), and E9. BDNF expression within the ganglia parallels that of TrkB: BDNF mRNA expression levels are highest at St 29/30 (E6.5), and these levels decrease 2-fold at St. 31 (E7) and St. 34 (E8; Figure 5). By E9, BDNF levels are 7 times lower than at St. 29/30 (E6.5; Figure 5).

### NT3 and NGF promote survival of differentiating sympathetic neurons in culture

To determine the effect of neurotrophins, we cultured cells dispersed from lumbar sympathetic ganglia at St. 29/30 (E6.5) because, at this stage, ganglion formation is complete, the number of TrkA-, TrkB-, and TrkC-positive cells have peaked, and all Trk-expressing cells have initiated neural differentiation. First, we identified markers expressed by acutely isolated cells. As shown in Table 1, 80–91% of the cells are p75 neurotrophin receptor (NTR)-positive, indicating that most of the cells are neural crest-derived and little mesenchymal contamination is introduced by the isolation procedure. In addition, 28–33% of the cultured cells express the neural marker Hu C/D. Approximately half of these Hu C/D-positive cells express TrkB. Conversely, all of the TrkB positive cells express Hu C/D. These TrkB-positive cells comprise approximately 14–17% of the total cell population.

**Table 1:** p75 NTR and Hu C/D Expression in Acutely Isolated Cells from St. 29/30 (E6.5) Sympathetic Ganglia

	Experiment 1 (mean values; n = 3)	Experiment 2 (mean values; n = 3)
p75NTR (% of total)	1974.3 +/- 368 (91%)	2128.7 +/- 490 (80%)
Hu C/D (% of total)	719.7 +/- 137 (33%)	751 +/- 47 (28%)
Total Number	2177.7 +/- 381	2637 +/- 701

Cells isolated from dispersed St 29/30 (E6.5) lumbar sympathetic ganglia were plated onto poly-lysine and laminin coated cover slips and allowed to attach for 2 hrs. Cover slips were then fixed and stained for p75 NTR and Hu C/D using double immunofluorescence. P75 NTR marks all cells derived from the neural crest, regardless of their state of differentiation. Hu C/D is a neural-specific RNA binding protein that is an early marker of neural differentiation. Each experiment is a separate plating and 3 cover slips were counted for each experiment.

We then determined how many of the acutely isolated cells were proliferating by incubating them for 12 hrs in BrdU-containing medium. For these experiments, we identified differentiating neurons with the transcription factor Islet-1 because this marker labels nuclei, thus it co localizes with any BrdU that has been incorporated into the DNA, allowing us to determine whether the cell had undergone S-phase of the cell cycle. After 12 hrs in BrdU, 59% of Islet-1-positive nuclei stain for BrdU immunoreactivity. Thus, cultures of St. 29/30 sympathetic ganglia contain many cells that proliferate while exhibiting markers of neuronal differentiation, confirming previous observations [2]. We call these dividing neuronal precursors sympathoblasts. The remaining non-BrdU incorporating, Islet-1 positive cells are likely to be post mitotic neurons.

**Figure 6:**
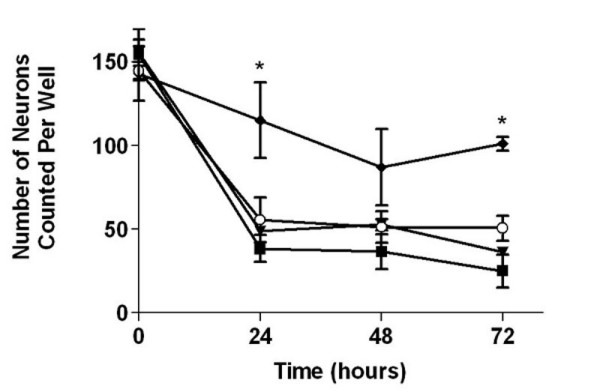
**St. 29/30 (E6.5) Sympathetic neurons require NT3 and NGF for survival**. St. 29/30 (E6.5) sympathetic ganglia were dissociated and sympathetic neurons were cultured as described in Methods. The cells were treated without neurotrophins (closed square), or with NT3 alone (open circle), NGF alone (closed triangle), or NT3 and NGF (closed diamond). The number of neurons in the same well was counted each day to assess survival. The neurons survive when cultured with both NT3 and NGF. Data represent mean ± SEM from three independent experiments. *Denotes statistical significance (p < 0.05), Two-way ANOVA.

Finally, we determined the trophic requirements of St. 29/30 (E6.5) sympathetic neurons and sympathoblasts. We monitored cultures over a three day period after plating and counted the number of phase bright cells with neurites, a morphological feature of both neurons and sympathoblasts. In the absence of trophic factors, more than 2/3 of the cells die by 24 hours in culture and BDNF, NT-3, or NGF alone is not sufficient to promote survival (Figure 6). However, NGF together with NT-3 supports the survival of a significantly larger number of cells (Figure 6). For the subsequent experiments, all neurons were cultured with 25 ng/ml NT-3 and 1 μg/ml 7S NGF to optimize survival.

### BDNF promotes proliferation of TrkB-positive sympathetic neurons in culture

**Figure 7:**
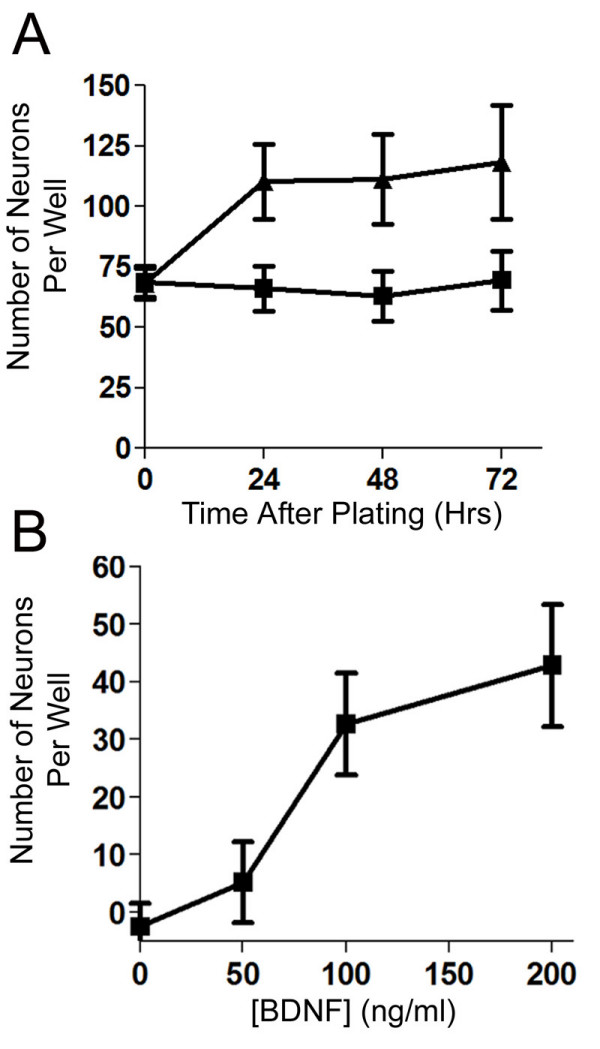
**BDNF increases the number of cells with neuronal morphology in cultures of dispersed St. 29/30 (E6.5) sympathetic ganglia**. (A) Cells from St. 29/30 (E6.5) lumbar sympathetic ganglia were cultured in control medium (closed squares) or control medium containing 200 ng/ml BDNF (closed diamonds) and the number of cells with neurites longer than two cell body diameters was scored under phase contrast optics at 24, 48, and 72 hours. A 1.6 fold increase in the number of neurons at each time point was seen with BDNF. Data represent mean ± SEM of six wells from two independent platings. *Denotes statistical significance (p < 0.05), Two-way ANOVA. (B) Cells from St 29/30 (E6.5) sympathetic ganglia were cultured with increasing concentrations of BDNF. The number of neurons was counted at the time BDNF was added to the cultures and 24 hours after BDNF was added to the cultures. Data correspond to the starting number of neurons subtracted from the number of neurons counted at 24 hours. Data represent the mean ± SEM of six wells from two independent platings.

To determine the effects of BDNF, cultures of cells from St. 29/30 (E6.5) sympathetic ganglia were supplemented with 200 ng/ml BDNF and the number of neurons and sympathoblasts were counted at 24, 48, and 72 hours using phase microscopy (Figure 7A). A 1.6-fold increase in the number of neurons due to BDNF is observed by 24 hours and this number does not increase further at 48 or 72 hours. This effect of BDNF is concentration-dependent with an EC_50 _of 75 ng/ml (Figure 7B).

**Figure 8:**
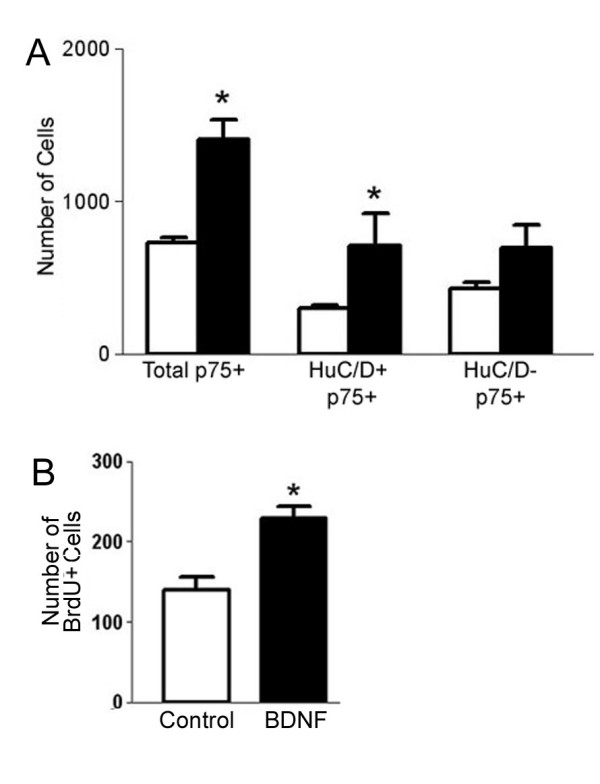
**BDNF increases the total number of differentiating neurons by inducing proliferation**. Cells from St. 29/30 (E6.5) lumbar sympathetic ganglia were cultured in control medium (white bars) or control medium containing 200 ng/ml BDNF (dark bars) for 24 hrs, then fixed and stained with anti-p75NTR and anti-Hu C/D. BDNF increases the total number of p75NTR-positive cells as well as the total number of neuronal (Hu C/D+), but not non-neuronal (Hu C/D-) cells. Data represent mean ± SEM of three cover slips for each condition. *Denotes statistical significance (p < 0.05), Student's t-test. (B) Cells were labeled with BrdU for the first 12 hours in culture, then fixed and stained for BrdU and Hoechst dye 24 hrs after plating. BDNF increases the number of BrdU-positive cells compared to control. Data represent mean ± SEM from six independent experiments. *Denotes statistical significance (p < 0.05, Students' t-test).

To test whether the increase in the number of neurons and sympathoblasts caused by BDNF is due to the differentiation of pluripotent neural crest cells, we quantified the effects of BDNF on the number of neurally differentiating cells (Hu C/D-positive) versus the number of non-neuronal cells (Hu C/D-negative) after identifying all neural crest-derived cells by staining for p75NTR in St. 29/30 (E6.5) cultures. If BDNF increases the number of neurons and sympathoblasts by inducing a non-neuronal cell to express Hu C/D, then we expected that the total cell number would remain the same and that there would be a decrease in the number of non-neuronal cells as well as a corresponding increase in the number of neurons. After 24 hours, BDNF significantly increases the number of p75NTR-positive cells as well as the number of Hu C/D-positive cells (Figure 8A). However, there was no statistically significant change in the number of non-neuronal cells. Thus, it is unlikely that BDNF increases the number of neurons and sympathoblasts by inducing differentiation of non-neuronal cells.

**Figure 9:**
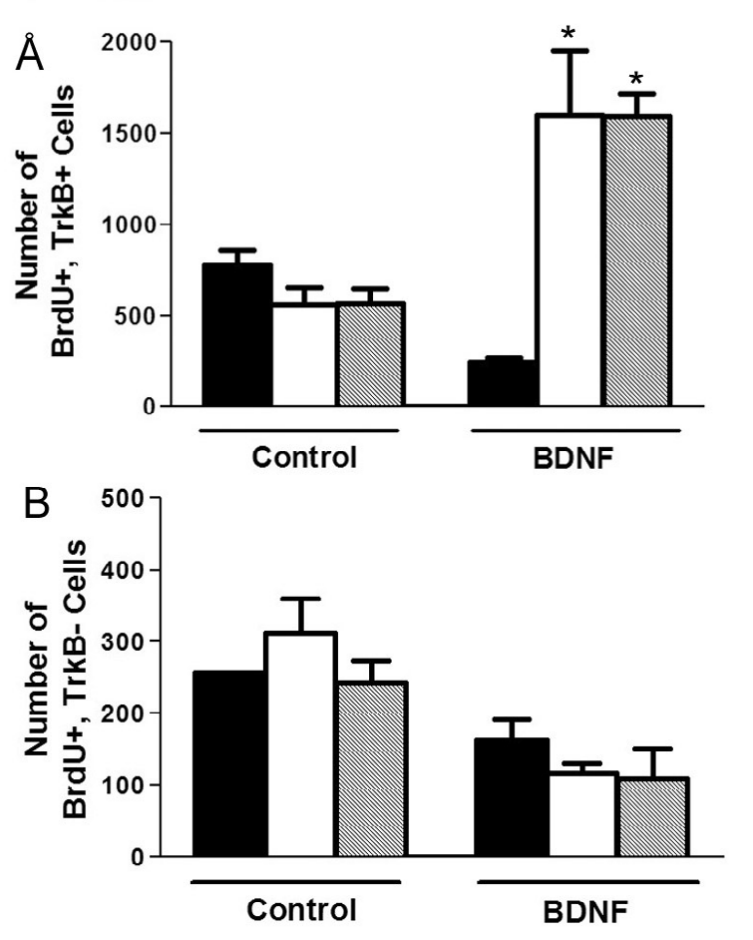
**The effects of BDNF are specific to TrkB expressing cells**. Cells from dispersed St 29/30 (E6.5) lumbar sympathetic ganglia were cultured in control medium or medium supplemented with 200 ng/ml BDNF together with 1:1000 rabbit anti-TrkB extracellular domain (dark bars), 1:1000 non-immune rabbit serum (white bars) or no addition (hatched bars). All cultures were pulsed for 12 hrs with BrdU, and then harvested 24 hrs after plating, fixed, and stained with anti-BrdU, anti-TrkB, and Hoechst dye. (A) BDNF induces a significant increase in the number of TrkB+, BrdU+ cells that are blocked by the antiserum against TrkB, but not by the non-immune rabbit serum. (B) In contrast, there is no increase in the number of TrkB-cells, and the antisera have no effect additional effects. The values in both (A) and (B) represent the mean ± SEM of 6 cover slips from two independent platings.

To determine whether the increase in the total number of neurons and sympathoblasts is caused by BDNF-induced proliferation, control and BDNF-treated sympathetic cultures were exposed to BrdU for 12 hours after plating, and the number of cells that incorporated BrdU into their DNA was determined after 24 hours in culture. Even in the control condition, a number of cells in the culture are dividing, giving a high baseline of BrdU incorporation (Figure 8B). When BDNF is added, the total number of BrdU positive cells increases approximately 1.6-fold (Figure 8B). This BDNF-induced increase in the total number of BrdU positive cells occurs in sympathoblasts because the number of Islet-1-positive nuclei from control cultures that label with BrdU is 268 ± 59 and BDNF treatment raises this number to 424 ± 80, which corresponds to an increase of 1.6-fold. This accounts for the 1.6-fold increase in total neuron number and total BrdU-positive cells described above. We then confirmed that BDNF acts on TrkB-positive cells: BDNF increases the number of TrkB-expressing cells that incorporate BrdU 2.6 – 4-fold over control (Table 2) and it also increases the overall number of TrkB-positive cells 2 – 2.5-fold over control (Table 2). BDNF does not increase the number of BrdU-positive, TrkB-negative cells or the overall number of TrkB-negative cells (Table 2). In further support that BDNF acts directly on TrkB-expressing cells, an antibody directed against the extracellular domain of TrkB completely prevents the effect of BDNF in promoting proliferation of TrkB-positive, but not TrkB-negative cells (compare Figure 9A to 9B). Thus, the effect of BDNF is restricted to the population that expresses TrkB, which are developing sympathoblasts.

**Table 2:** Effects of BDNF on BrdU Incorporation in TrkB-Expressing Cells from St. 29/30 (E6.5) Sympathetic Cultures

	Control	BDNF	Fold Increase
Experiment 1:			
BrdU+/TrkB+	219 ± 34	845* ± 95	4×
TrkB+	438 ± 55	1111* ± 153	2.5×
BrdU+/TrkB-	258 ± 36	187 ± 14	No change
TrkB-	962 ± 42	900 ± 109	No change

Experiment 2:			
BrdU+/TrkB+	258 ± 54	673* ± 64	2.6×
TrkB+	508 ± 77	947* ± 126	2×
BrdU+/TrkB-	211 ± 14	180 ± 48	No change
TrkB-	892 ± 75	834 ± 105	No change

Cultures of St 29/30 (E6.5) lumbar sympathetic ganglia were exposed to 10 μM BrdU for 12 hrs, followed by complete medium lacking BrdU. Twenty-four hrs after plating, cells were fixed and stained for TrkB and BrdU using double immunofluorescence. All nuclei were also stained with Hoechst dye. The total number of BrdU+, TrkB+ and BrdU+, TrkB-cells was scored by counting 10 fields of view using epifluorescence optics. In experiments 1 and 2 respectively, 76% and 71% of the TrkB+ cells incorporate BrdU in response to BDNF. Each experiment represents the mean +/- SEM of 3 cover slips from two independent platings.

**Figure 10:**
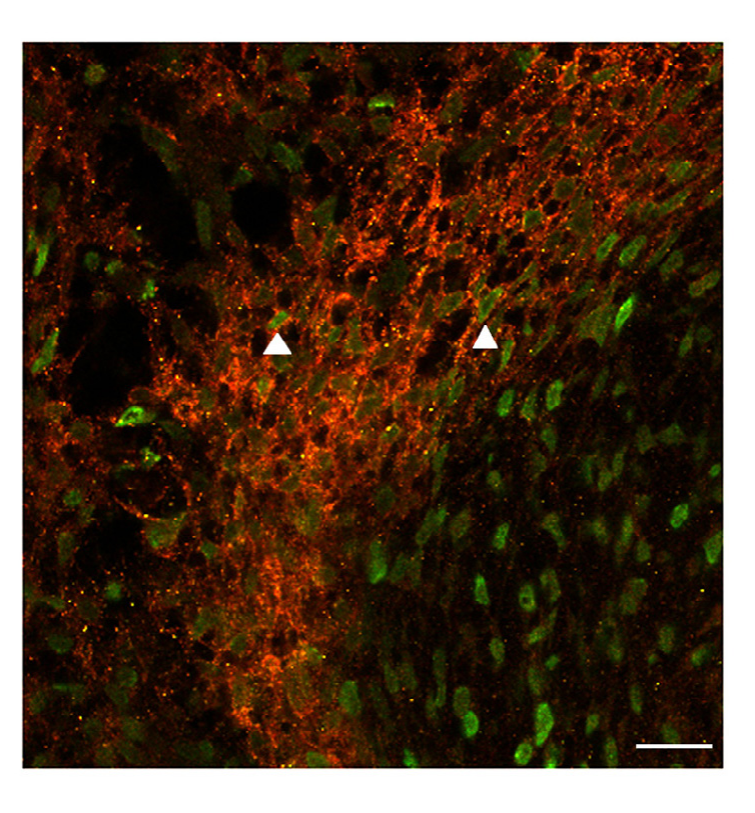
**The majority of TrkB-positive cells in St. 29 sympathetic ganglia do not proliferate under basal conditions *in vivo***. St. 27 embryos were injected with 25 μg BrdU and embryos were harvested at St. 29. Transverse sections of sympathetic ganglia prepared to determine whether TrkB-positive cells (red) in sympathetic ganglia positively-labeled for BrdU. The majority of TrkB-positive cells do not proliferate under basal conditions *in vivo*. Arrows indicate proliferating cells. Calibration bars = 20 μ.

To determine whether TrkB-positive cells are actively proliferating *in vivo*, embryos were injected with BrdU at St. 27 and harvested at St. 29, approximately 24 hrs later. The majority (85–90%) of TrkB-positive cells do not incorporate BrdU into their nuclei under basal conditions *in vivo *(Figure 10), although a few TrkB positive cells with labeled nuclei could be observed (arrows). This contrasts with our observation that 71–76% of TrkB-positive cells incorporate BrdU in culture after treatment with BDNF (Table 2), suggesting that endogenous BDNF does not achieve a threshold sufficient to support a high level of sympathoblast proliferation *in vivo*.

## Discussion

We report that the neurotrophin receptor TrkB is expressed in a subset of embryonic sympathoblasts during the early development of lumbar paravertebral sympathetic ganglia in chicken and mouse embryos. In the chicken, TrkB expression is transient, and completely lost by St 34 (E8). Since BDNF induces the proliferation of sympathoblasts in cell culture, yet *in vivo *there is little proliferation observed in TrkB-positive cells in nascent ganglia, we propose that if TrkB activation becomes unregulated by excess BDNF or constitutive phosphorylation of TrkB [16], this transient population of TrkB-positive sympathoblasts may trigger the genesis of neuroblastoma, a childhood tumor found in the paravertebral chain and adrenal medulla.

The two populations of sympathoblasts that we observe support previous findings of heterogeneity among developing sympathetic neurons and neural crest cells. Early sympathetic ganglia contain at least two subpopulations: early differentiating neurons that lack TrkB expression and express TrkA and TrkC, and late differentiating sympathoblasts that express TrkB. Explant cultures of sympathetic ganglia from E16 chick embryos give rise to two neuronal populations: one that remains close to the explant, and one that migrates away from the explant [1]. In addition, early neuronal subpopulations have been observed in cultures of neural crest cells from St. 13/14 quail embryos as evidenced by the expression of neuronal cell type-specific gangliosides [17]. Perhaps these different subpopulations will ultimately give rise to the two neurochemically distinct populations found in lumbar sympathetic ganglia: the noradrenergic, NPY-containing neurons that innervate internal organs and enteric ganglia and the cholinergic, VIP-containing neurons that innervate vasculature in the hind limbs.

The effects of BDNF and TrkB deletion and over expression have been studied on superior cervical ganglion and preganglionic neurons in thoracic segments of the spinal column, but not on paravertebral sympathetic neurons. In the superior cervical ganglion, an increase in the number of neurons of BDNF null mice is likely due to apoptosis induced by BDNF via p75NTR [18]. In contrast, the responses of paravertebral sympathetic neurons to BDNF are complex and subtype dependent. Over expression of BDNF leads to an increase in the number of noradrenergic fibers innervating the erector pilli muscles of hair follicles, while noradrenergic fibers innervating blood vessels were unaffected [19]. If our results indicating that BDNF promotes proliferation of TrkB-positive sympathoblasts in the chicken embryo can be extrapolated to the subset of TrkB-positive sympathoblasts in murine ganglia, then these TrkB-positive cells may be neurons destined to innervate the erector pilli. In other studies, TrkB null mice showed no changes in morphology or cell number in superior cervical ganglia [12] or in the intermediolateral column [20]; but this may not be predictive of a phenotype in the lumbar paravertebral chain. It is thus possible that BDNF/TrkB signaling could play a specific role in other regions of the paravertebral sympathetic chain, such as the lumbar region. However, if TrkB-positive cells are not normally actively proliferating *in vivo*, then it would not be surprising that the development of the paravertebral sympathetic chain is not disrupted in TrkB or BDNF null mice. It may be more informative to examine mice that over express BDNF on a promoter that targets expression to embryonic lumbar ganglia. Unfortunately, such mice do not exist.

Our findings that the St. 29/30 (E6.5) sympathoblasts are dependent on both NT-3 and NGF for survival in culture are consistent with previous work on mouse sympathoblasts from the superior cervical ganglion [11]. In these studies, NT-3 and NGF deletion separately led to a decrease in the number of sympathetic neurons at E17.5 compared to control. Deletion of both NT-3 and NGF together did not enhance cell death. In contrast, cultured rat superior cervical ganglion sympathetic neurons respond to NT-3 at E14.5 and then to NGF at E19.5, although time points in between were not analyzed [6].

In addition to promoting survival, NT-3, NGF, and BDNF also induce proliferation of various neuronal precursors at different stages of development. NT-3 can promote the incorporation of [^3^H]-thymidine into cultured quail neural crest cells from the trunk region [21,22], Later in rat sympathetic development, NT-3 supports survival of neurons, but does not promote proliferation [6], which is consistent with our results. NGF promotes an increase in BrdU incorporation from 25% to 35% in the DRG cervical segment 2 in the chick embryo [23]. In chicken embryos that are treated with NGF *in ovo *at St. 18 and 21, there is an increase in BrdU uptake after formation of the primary sympathetic chain at St. 23 [24]. Since NGF does not appear to affect proliferation of St. 29/30 (E6.5) chick sympathoblasts, NGF may only promote proliferation in primary, but not secondary chain sympathoblasts. Motor neuron progenitors in the ventral neural tube from the chick embryo express TrkB and when ventral neural tube explants are treated with BDNF, there is an increase in the number of motor neurons produced and BrdU incorporation [25]. BDNF also promotes the proliferation of cultured neuroblastoma cells [13]. Taken together, these results are consistent with our findings that NT-3 and NGF do not promote proliferation of St. 29/30 (E6.5) sympathoblasts, and support the assertion that BDNF promotes proliferation of TrkB-positive sympathoblasts in culture.

Our observations suggest a transient function of TrkB during early sympathetic development in supporting proliferation of this early subpopulation of sympathoblasts. However, the *in vivo *labeling suggests that only a minority (10–20%) of this population is dividing during the window that TrkB is expressed. In light of the very strong proliferative effect produced in cell culture, these TrkB expressing cells could respond more strongly if endogenous BDNF rises to higher levels, or if the mechanism that down regulates TrkB expression becomes nonfunctional. Such events could trigger an early proliferative event that leads to a cascade of changes that initiates transformation of cells to neuroblastoma. Thus, these early TrkB expressing cells help solve the puzzle as to why TrkB is expressed in aggressive and invasive forms of neuroblastoma, particularly because BDNF induces cultured neuroblastoma cells to become more proliferative, invasive, angiogenic, and resistant to chemotherapeutic reagents than untreated cultures [13]. Future studies will determine whether constitutive expression of BDNF and TrkB in the chick embryo sustains proliferation of differentiating sympathoblasts.

## Conclusion

We have identified a time point during development when differentiating lumbar sympathetic neurons transiently express TrkB and proliferate in response to high concentrations of BDNF in culture. These studies suggest that elevated BDNF expression above basal levels and signaling through TrkB may be a mechanism that contributes to the onset of neuroblastoma. A further understanding of the two populations of sympathetic neurons and the fate of the TrkB-positive cells will provide additional insight into the development of paravertebral sympathetic ganglia and the genesis of neuroblastoma.

## Methods

### Preparation of tissue for immunohistochemistry

The lumbar spinal column and surrounding tissues were dissected from chicken embryos at the indicated stages and placed in Zamboni's fixative (4% (w/v) paraformaldehyde, 15% (v/v) picric acid in 0.1 M sodium phosphate buffer, pH 7.4) for two hours at room temperature. Mouse embryos at 13–15 days post-coitus were collected according to an IACUC-approved protocol to Dr. L. Sherman at the Oregon Health and Science University. The mouse embryos were immersion-fixed in Zamboni's fixative overnight at 4 degrees C then washed with phosphate buffered saline (PBS; 130 mM NaCl, 20 mM sodium phosphate buffer, pH 7.4). Fixed tissues were equilibrated in 30% sucrose in 1× phosphate-buffered saline (PBS). Fixed mouse embryos were shipped to Vermont in sucrose. Transverse 30 μM sections of the spinal columns were cut at on a Microm HM cryostat (knife temperature: 16°C; object temperature: 23°C) and collected on Superfrost Plus slides (Fisher). Sections were dried at room temperature, washed in 1× PBS and incubated overnight in blocking buffer (1× PBS consisting of 10% (v/v) heat-inactivated horse serum (Invitrogen/Gibco), 0.5% Triton X-100 (Sigma), and 0.1% sodium azide (Fisher)).

### Immunohistochemistry

Sections were incubated with primary antibodies overnight at 4°C, followed by incubation with secondary antibodies for 2 hours at room temperature. Primary antibodies used were: rabbit anti-p75 (1:1500, generous gift from Louis Reichardt, UCSF [26]), mouse IgG_2b _anti-Hu C/D, (1:250, Molecular Probes); mouse IgG_1 _anti-Islet-1, (1:10, Developmental Studies Hybridoma Bank); rabbit anti-chicken TrkA (1:500); rabbit anti-chicken TrkB (1:500); rabbit anti-chicken TrkC (1:500) (all Trk antibodies were generous gifts of Dr. Louis Reichardt, UCSF [26-28]); mouse anti-HNK-1 (1:50, Developmental Studies Hybridoma Bank); mouse IgG_2a _anti-tyrosine hydroxylase (1:10, Developmental Studies Hybridoma Bank), sheep anti-BrdU (1:100, Biodesign International), rabbit anti-tyrosine hydroxylase (1:100, Chemicon), and goat anti-TrkB (1:1000, R&D Systems). Immunofluorescence was imaged using a Nikon C1 confocal mounted on a Nikon Eclipse E800 microscope with a 10× Plan Apo (NA 0.785) air objective or a 60× Plan Apo (NA 1.4) oil objective lens, E7-C1 software, and UV, Argon, and He/Ne lasers exciting at 408, 488, and 543 nm and emitting at 404 500–530, and 555–615 nm, respectively. A Nikon Eclipse E800 microscope in the nearby COBRE Molecular/Cellular Core Facility was used for counting immunofluorescent cells at 200× using epifluorescence optics.

### RNA Extraction/cDNA synthesis

Sympathetic ganglia were removed from chick embryos and RNA was isolated using TriReagent (Molecular Research Center), an acidified guanidinium with phenol extraction method [29]. RNA was transcribed to cDNA using oligo-dT with Superscript II Reverse Transcriptase (Invitrogen) at 42°C for 1 hour.

### Real-time PCR

Relative RNA levels were determined using quantitative real-time PCR with an ABI 7500 Fast Real Time PCR System. TaqMan probes were used to quantify the progression of the PCR reaction and reactions were normalized using the constitutively expressed gene chick ribosomal binding protein s17 (CHRPS). The sequences were used for primer/probes sets: for BDNF: forward: 5'-AGCCCAGTGAGGAAAACAAG-3', reverse: 5'-ACTCCTCGAGCAGAAAGAGC-3', probe: 5'-[6-FAM]-TACACATCCCGAGTCATGCTGAGCA-[BHQ]-3'; for CHRPS (chick ribosomal binding protein S-17): 5'AACGACTTCCACACCAACAA3', reverse: 5'CTTCATCAGGTGGGTGACAT3', probe: 5'-[6-FAM]-CGCCATCATCCCCAGCAAGA [BHQ]-3'. Primers and probes were synthesized by Operon Technologies, Inc (Alameda, CA). The primers for BDNF were validated against primers for CHRPS according to an Applied BioSystems protocol by serially diluting the target cDNA 1:10, determining the cycle threshold (C_t_) for each reaction, and plotting the C_t _versus log concentration. Slopes of the resulting lines were calculated and primers were accepted if their C_t _slopes were between -3.2 and -3.4 (a perfect efficiency of 1.0 yields a slope of -3.3). To analyze the data, the delta C_t _method of relative quantification was used, where the C_t _of Chrps was subtracted from the C_t _of the gene of interest (Delta C_t_) and the arbitrary units of mRNA were expressed as 10000/2^(Delta C_t_).

### Cell culture

Sympathetic neurons were cultured as previously described [30] with a few modifications. Sympathetic ganglia were removed from the lumbar region of the paravertebral chain of St. 29/30 (E6.5) chick embryos and placed in Modified Puck's solution with glucose (MPG). The cells were dissociated by incubation of sympathetic ganglia with 0.1% trypsin in MPG at 37°C for 10 minutes followed by triturating with a fire polished 9" Pasteur pipette. Cells were then resuspended in Dulbecco's Modified Eagle Medium (DMEM) consisting of 10% horse serum, 2% fetal calf serum, and 10 mg/ml penicillin/streptomycin. For neurotrophin studies, the culture medium was supplemented with 25 ng/ml NT-3 (R & D Systems) and 1 μg/ml 7S NGF (Alomone Labs) upon plating, and 50 ng/ml, 100 ng/ml, or 200 ng/ml BDNF (R & D Systems) once the cells adhered to the wells. Cells were plated on poly-D-lysine/laminin coated wells or cover slips (Fisher) as previously described [30].

### Quantification of neurons and sympathoblasts using phase microscopy

Embryonic sympathoblasts and neurons are small, phase bright cells with neurites. The total number of cells with neurites the length of two cell bodies were counted in 10 non-overlapping fields of view evenly spaced in a grid-like pattern across the bottom of a well from a 24 well plate at 200× using a Nikon Eclipse TE200 microscope.

### BrdU labeling

For *in vitro *studies, approximately 2 hours after plating cells from St. 29/30 (E6.5) sympathetic ganglia, cells were labeled with 10 μM bromodeoxyuridine (BrdU, Sigma) for 12 hours at 37°C. Following this labeling period, cells were incubated in complete medium without BrdU for an additional 10 hrs. Cells were then fixed in Zamboni's fixative for 30 min at room temperature and rinsed with 1× PBS. For *in vivo *studies, 25 μg BrdU was injected into the amnion of chick embryos at St. 27. The cells and sections were denatured with 2 N HCl at 37°C for 1 hr, and were then neutralized with 0.1 M borate buffer, pH 8.5, for 10 min at room temperature. Immunochemistry was performed as described above.

## Abbreviations

BDNF, brain-derived neurotrophic factor; BrdU, Bromodeoxyuridine; DA, dorsal aorta; DMEM, Dulbecco's Modified Eagle's Medium; DRG, dorsal root ganglion; E, embryonic day; HS, horse serum; MPG, Modified Puck's solution with glucose; NGF, nerve growth factor; NC, notochord; NT, neural tube; NT-3, neurotrophin-3; NTR, neurotrophin receptor; PBS, phosphate-buffered saline; SC, spinal cord; SCG, superior cervical ganglion; SEM, standard error of the mean; SG, sympathetic ganglion; St., stage; w/v, weight/volume; v/v, volume/volume.

## Authors' contributions

JAS designed the experiments, performed the experiments, analyzed the data, and wrote the manuscript. GLSS contributed intellectually to the conception and design of this study, and assisted in the interpretation of the results. RN supervised the study, participated in the design of experiments, edited the manuscript, and obtained funding for the project. All authors read and approved the final manuscript.
